# Systemic Lupus Erythematosus Patients With Related Organic Damage Are at High Risk of Hypothyroidism

**DOI:** 10.3389/fendo.2022.920283

**Published:** 2022-07-15

**Authors:** Jiajia Ni, Jingyi Li, Yuyao Wang, Liying Guan, Haiyan Lin, Li Zhang, Haiqing Zhang

**Affiliations:** ^1^ Department of Endocrinology, Shandong Provincial Hospital, Shandong University, Jinan, China; ^2^ Department of the Health Management Center, Shandong Provincial Hospital Affiliated to Shandong First Medical University, Jinan, China; ^3^ Department of Vascular Surgery, Shandong Provincial Hospital Affiliated to Shandong First Medical University, Jinan, China

**Keywords:** hypothyroidism, systemic lupus erythematosus, lupus nephritis, liver abnormality, cardiac insufficiency

## Abstract

**Purpose:**

The aim of this study included determining the prevalence of hypothyroidism in patients with systemic lupus erythematosus (SLE), clarifying the clinical characteristics of SLE patients with hypothyroidism, and identifying the relationship between hypothyroidism and SLE-related organic damage. Another purpose was to analyze the relationship between SLE and thyroid autoantibody. We also intended to discuss the pathogenesis of hypothyroidism in SLE patients, which would provide clues for further investigation.

**Methods:**

This study recruited 856 SLE patients and 856 age- and sex-matched healthy population and compared the prevalence of hypothyroidism between the cases and controls. Univariate and multivariate logistic analyses were applied to identify risk factors for hypothyroidism in SLE patients.

**Results:**

SLE patients had higher prevalence of clinical hypothyroidism (9.10%) and TgAb+TPOAb- (10.40%) than controls. The prevalence of hypothyroidism was the highest in SLE patients aged 16-26 years (18.9%) and decreased with age. The prevalence of autoimmune hypothyroidism in SLE group was higher than that in the control group (64.4% vs. 51.5%, *P*=0.042), which was mainly due to TgAb; the prevalence of non-autoimmune hypothyroidism in SLE group was also significantly higher than that in the control group (67.3% vs. 47.8%, *P*<0.001). Based on multivariate analysis, the use of glucocorticoids/immunosuppressants, liver abnormality, lupus nephritis (LN), and cardiac insufficiency were independently associated with hypothyroidism in SLE patients.

**Conclusion:**

The prevalence of hypothyroidism in SLE patients was higher than that in controls and decreased with age. The results suggested that young SLE patients combined with LN, liver abnormality and cardiac insufficiency were at higher risk of hypothyroidism. According to the results of this study, we speculated that SLE might have impact on thyroid, and SLE might be one of the causes of hypothyroidism.

## Introduction

Systemic lupus erythematosus (SLE) is a severe systemic autoimmune disease, which involves multiple organs, including kidney, liver, joints, blood, skin, vessels, nervous system, lung, and heart. The prevalence of SLE ranges from 19 to 159 per 100,000 (1–5). Due to the advancement in diagnosis and treatment, the mortality rate of SLE has been greatly reduced. However, the mortality rate in SLE patients was still three times higher than that in the general population (6). Autoimmune thyroiditis (AIT) is a common autoimmune thyroid disease, characterized by infiltration of immune cells in thyroid tissue, which is the most common etiology of hypothyroidism (7). The prevalence of hypothyroidism in Chinese general population was approximately 13.95% (8). Due to common genetic and gender background, it is believed that SLE has association with other autoimmune diseases. One study indicated that Sjogren syndrome (SS) and AIT were “chaperones” of SLE (9). Hypothyroidism is also the most frequent thyroid dysfunction in SLE. According to previous studies, the prevalence of hypothyroidism in SLE varied from 15% to 19%, and it was higher than that in the general population (10). In addition, some clinical evidence revealed that there was a certain relationship between the severities of these two diseases (11, 12). Although hypothyroidism is frequent in SLE, the examination principle of SLE patients is still the same as that of general population. Meanwhile, atypical clinical symptoms of hypothyroidism are easy to be covered by the clinical manifestations of SLE, which can lead to the delay of diagnosis and treatment of hypothyroidism in SLE patients. Currently, few studies have been conducted on clinical features of SLE patients with hypothyroidism.

There were plentiful researches on SLE and hypothyroidism, but most of them only spotted the light on the prevalence of hypothyroidism. Few studies focused on the relationship between hypothyroidism and SLE-related organic damage. However, our study performed more comprehensive analyses with larger sample size and more standard control group. In addition, thyroid autoantibodies were involved in this study. To put it in a nutshell, this study aims to disclose and demonstrate the prevalence of hypothyroidism and the clinical characteristics in SLE patients with hypothyroidism, and recognize the relationship between hypothyroidism and SLE-related organic damage. More promisingly, this work also discussed the potential pathogenesis of hypothyroidism in SLE patients, which would provide implications and suggestions for further investigation.

## Methods

### Patients

**Figure 1:**
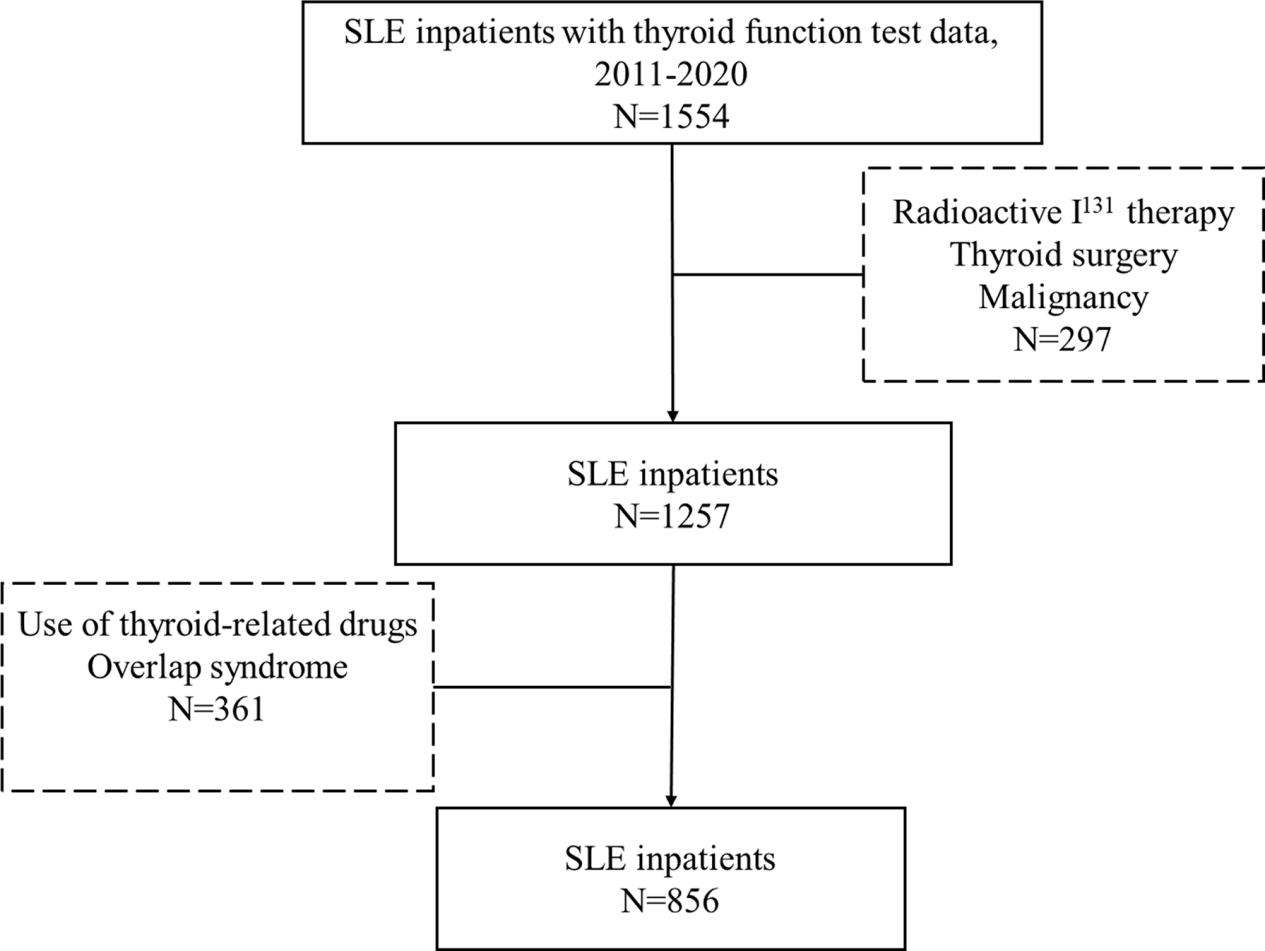
The inclusion and exclusion criteria of SLE patients.

This study was a retrospective study, which recruited a total of 856 SLE patients who were admitted to Shandong Provincial Hospital from November 2011 to July 2020. All subjects met the American College of Rheumatology Association (ACR) 1997 revised SLE classification criteria (13). The study had also included these patients who were newly diagnosed or under treatment. We enrolled 1554 SLE patients who were hospitalized in the Department of Rheumatology and Immunology of Shandong Provincial Hospital from 2011 to 2020 and underwent thyroid function examination. After careful and precise consideration, we screened out and excluded patients with treatment of the I^131^, thyroid surgery, and thyroid-related drugs and patients with history of malignance and overlap syndrome. The inclusion and exclusion criteria were listed in **Figure 1**. The study was approved by the Medical Ethics Committee of Shandong Provincial Hospital, Jinan, Shandong, China (NO. SWYX2020-187).

### Controls

The control group included 856 subjects performed health checks at the Health Management Center, Shandong Provincial Hospital from 2016 to 2019, and frequently matched to cases regarding sex and age. The exclusion criteria included thyroidectomy, radioactive I^131^ therapy, use of thyroid-related drugs, malignancy, and autoimmune disease.

### Classification of Thyroid Diseases

According to the thyroid function, euthyroidism and hypothyroidism were diagnosed as follows: 1) Euthyroidism: normal thyroid-stimulating hormone (TSH) levels with normal free thyroxine (FT4) levels and free tri-iodothyronine (FT3) levels, and without thyroid autoantibodies; 2) Hypothyroidism: high TSH levels with low or normal thyroid hormone levels; 3) Clinical hypothyroidism (C-hypothyroidism): high TSH levels with low FT4 or FT3 levels; 4)Subclinical hypothyroidism (S-hypothyroidism): high TSH levels with normal FT4 or FT3 levels; 5): Autoimmune hypothyroidism (AH), hypothyroidism and TgAb positivity or/and TPOAb positivity; 6): Non-Autoimmune hypothyroidism (NAH), hypothyroidism and negative thyroid autoantibodies. The reference ranges for FT4, FT3, TSH, TgAb, and TPOAb are 12.0−22.0 pmol/L, 3.1−6.8 pmol/L, 0.27−4.20 µIU/ml, 0−115 IU/ml, and 0−34 IU/ml, respectively. Thyroid function and autoantibodies were detected by electrochemiluminescence (Roche, Cobas e601).

### Clinical Variables

We collected the patients’ medical records. Lupus nephritis (LN) was diagnosed according to 2012 Systemic Lupus International Collaborating Clinics (SLICC) classification criteria (14). The presence of anemia (hemoglobin<10 g/dL) and positive Coombs test were the diagnosis criteria for hemolytic anemia (13). Patients were diagnosed as liver abnormality when liver enzymes were elevated with immunologic hepatitis or drug-related causes excluded. Patients with LVEF ≤ 45% were diagnosed as cardiac insufficiency. Ground-glass appearance in high-resolution computed tomography (HRCT) was helpful to diagnose interstitial lung pneumonia (ILD). Doppler echocardiography to look for elevations in estimated pulmonary artery pressure and resistance and/or tricuspid valve insufficiency was the diagnostic test for pulmonary hypertension (PH). The diagnosis of neuropsychiatric SLE (NPSLE) met the ACR 1999 revised nomenclature and case definitions for neuropsychiatric lupus syndromes (15). Patients were diagnosed as serositis when pleural effusion, pericardial effusion, or multiple serosal cavity effusion were noted in the discharge diagnosis. We also collected information about the use of glucocorticoids/immunosuppressants within three months before hospitalization.

### Laboratory Variables

The erythrocyte sedimentation rate (ESR), complement 3 (C3), complement 4 (C4), antinuclear antibody (ANA), anti-double-stranded deoxyribonucleic acid antibodies (anti-dsDNA), anti-Sjogren’s syndrome type A antibodies (anti-SSA), anti-Sjogren’s syndrome type B antibodies (anti-SSB), anti-Smith antibodies (anti-SM), creatinine, 24h urine protein, albumin, triglyceride, high density lipoprotein (HDL), and diastolic blood pressure were collected from medical documents. The reference values for the ESR, C3, C4, ANA, anti-dsDNA, anti-SSA, anti-SSB, anti-SM, creatinine, 24h urine protein, albumin, triglyceride, HDL, and diastolic blood are 0−20 mm/h, 0−10 mg/L, 0.9−1.8 U/ml, 0.1−0.4 IU/ml, >1:100, 0−100 IU/ml, 0−20 RU/ml, 0−20 RU/ml, 0−20 RU/ml, 40−105 mmol/L, 0−0.15 g/d, 40−55 g/l, 0.4−1.8 mmol/L, 0.8−1.5 mmol/L, and 60−90 mmHg, respectively. Indirect immunofluorescence was applied to detect ANA and anti-dsDNA. Enzyme-linked immunosorbent assay (ELISA) was used to spot anti-SSA and anti-SSB. Anti-SM was checked by chemiluminescent immunoassay.

### Statistical Analysis

Continuous variables were expressed as the mean (M) ± standard deviation (SD). Categorical variables were expressed by percentages. Categorical variables were compared by the chi-square test. Continuous variables were compared by t-tests. Binomial logistic regression analysis was used for univariate or multivariate analysis. SPSS version 25 was applied in statistical analysis. *P*<0.05 indicated that the difference was statistically significant.

## Results

### Characteristics of the SLE and Control Group

The proportion of females was 91.70% in SLE patients and controls. The average ages of SLE patients and controls were 41.11 years old and 42.29 years old, respectively (**Table 1**).

**Table 1:** Characteristics and prevalence of thyroid disease in the SLE and control groups.

	SLEN=856	ControlN=856	*P*	OR
Age M ± SD (y)	41.11 ± 14.28	42.29 ± 11.92	0.064	
Female n/N (%)	785/856 (91.70)	785/856 (91.70)	>0.999	1.000
Hypothyroidism n/N (%)	132/856 (15.40)	52/856 (6.10)	**<0.001**	2.113
C-Hypothyroidism n/N (%)	78/856 (9.10)	6/856 (0.70)	**<0.001**	14.203
S-Hypothyroidism n/N (%)	54/856 (6.30)	46/856 (5.40)	0.410	1.186
AH n/N (%)	56/856 (6.50)	31/856 (3.60)	**0.008**	1.863
TgAb+TPOAb- n/N (%)	89/856 (10.40)	64/856 (7.50)	**0.034**	1.436
TgAb-TPOAb+ n/N (%)	34/856 (4.00)	38/856 (4.40)	0.630	0.890
TgAb+TPOAb+ n/N (%)	107/856 (12.50)	93/856 (10.90)	0.292	1.172

y, year; SD, standard deviation; TgAb+, thyroglobulin antibodies positivity; TPOAb+, anti-thyroperoxidase antibodies positivity; TgAb-, thyroglobulin antibodies negativity; TPOAb-, anti-thyroperoxidase antibodies negativity.

Bold values are statistical differences p < 0.05.

### Prevalence of Hypothyroidism and Thyroid Autoantibody in SLE and Control Groups

**Figure 2:**
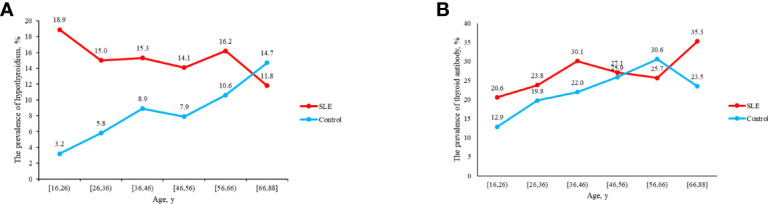
**(A)** The prevalence of hypothyroidism in SLE and control groups as age increases. **(B)** The prevalence of thyroid antibody in SLE and control groups as age increases.

**Figure 3:**
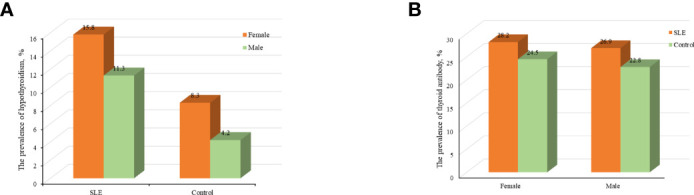
**(A)** Analyses of the prevalence of hypothyroidism in SLE and control groups according to sex. **(B)** Analyses of the prevalence of thyroid antibody in SLE and control groups according to sex.

**Figure 4:**
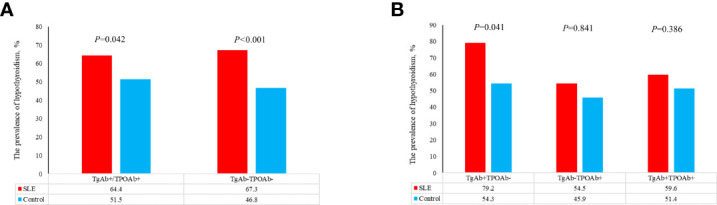
**(A)** The prevalence of hypothyroidism in patients with different thyroid antibodies in SLE group and control group. **(B)** The prevalence of hypothyroidism in patients with different thyroid antibodies in SLE group and control group.

Compared with control group, SLE patients had a higher prevalence of C-hypothyroidism (9.10% vs. 0.10%, *P*<0.001), AH (6.50% vs. 3.60%, *P*=0.008), TgAb^+^TPOAb- (10.40% vs. 7.50%, *P*=0.034). No significant difference in TgAb-TPOAb^+^ was found between the SLE patients and controls (**Table 1**). With the increase of age, the prevalence of hypothyroidism in SLE patients decreased gradually, while the trend of the general population was opposite. For the population younger than 65 years old, the prevalence of hypothyroidism in SLE patients was higher than that in the general population. However, with the increase of age, the difference between these two groups gradually decreased (**Figure 2A**). The prevalence of hypothyroidism and thyroid autoantibody in SLE and control group did not have significant difference between male and female (**Figure 3**). The prevalence of hypothyroidism with positive thyroid autoantibodies in SLE group was slightly higher than that in control group (64.4% vs. 51.5%, *P*=0.042), mainly due to TgAb; the prevalence of hypothyroidism with negative thyroid autoantibodies in SLE group was significantly higher than that in the control group (67.3% vs. 47.8%, *P*<0.001) (**Figure 4**).

### Comparison Study of Laboratory and Clinical Variables in SLE Patients With Euthyroidism and Hypothyroidism

SLE-hypothyroidism patients had a lower rate of the use of glucocorticoids/immunosuppressants treatment within three months before hospitalization, higher level of ESR, creatinine, 24h urine protein, triglyceride and diastolic blood, lower level of C3, albumin and HDL, and a higher rate of low C4 than SLE-euthyroidism patients (**Table 2**). Regarding organic damage, patients with SLE-hypothyroidism had a higher prevalence of liver abnormality, LN, serositis, hemolytic anemia, and cardiac insufficiency than patients with SLE-euthyroidism (**Table 2**).

**Table 2:** Comparison of serological and clinical profiles in SLE patients with euthyroidism or hypothyroidism.

	SLE-Hypothyroidism	SLE-Euthyroidism	*P*
**Epidemiological data**			
N	132	198	
Age (y) M ± SD	39.94 ± 14.55	40.91 ± 13.74	0.538
Female n/N (%)	124/132 (93.90)	177/198 (89.40)	0.219
**Serological profile**			
ESR (mm/h) M ± SD	43.63 ± 31.00	32.32 ± 24.49	**0.001**
C3 (g/l) M ± SD	0.70 ± 0.34	0.83 ± 0.29	**0.001**
Low C4 n/N (%)	55/109 (50.50)	58/160 (36.30)	**0.020**
ANA n/N (%)	93/94 (98.90)	155/157 (98.70)	>0.999
Anti-dsDNA n/N (%)	44/96 (45.80)	69/157 (43.90)	0.770
Anti-SSA n/N (%)	46/89 (51.70)	68/146 (46.60)	0.447
Anti-SSB n/N (%)	7/89 (7.90)	13/143 (9.10)	0.934
Anti-SM n/N (%)	23/83 (27.70)	45/122 (36.90)	0.223
Creatinine (mmol/L) M ± SD	74.90 ± 68.40	59.05 ± 45.36	**0.025**
24 h urine protein (mg/d) M ± SD	1.67 ± 2.71	0.35 ± 0.81	**<0.001**
Albumin (g/l) M ± SD	32.17 ± 8.39	38.20 ± 5.70	**<0.001**
Triglycerides (mmol/l) M ± SD	2.55 ± 1.51	1.64 ± 0.84	**<0.001**
HDL (mmol/l) M ± SD	1.10 ± 0.43	1.34 ± 0.48	**0.004**
Diastolic pressure (mmHg) M ± SD	81.71 ± 13.86	78.30 ± 12.62	**0.026**
**Organic damage**			
Treatment ^a^ n/N (%)	54/132 (40.70)	141/198 (71.20)	**<0.001**
Liver abnormality n/N (%)	33/132 (25.00)	23/198 (11.60)	**0.002**
Serositis n/N (%)	23/132 (17.40)	17/198 (8.60)	**0.025**
LN n/N (%)	52/132 (39.40)	34/198 (17.20)	**<0.001**
Hemolytic anemia n/N (%)	5/132 (3.80)	1/198 (0.50)	**0.040**
Cardiac insufficiency n/N (%)	12/132 (9.10)	2/198 (1.00)	**0.001**
ILD n/N (%)	12/132 (8.70)	11/198 (5.40)	0.335
PH n/N (%)	7/132 (5.30)	6/198 (3.00)	0.453
NPSLE n/N (%)	9/132 (6.80)	6/198 (3.00)	0.177

a Treatment: the use of glucocorticoids/immunosuppressants within three months before hospitalization.

Bold values are statistical differences p < 0.05.

### Comparison Study of Laboratory and Clinical Variables in SLE-C-Hypothyroidism Patients and SLE- S-Hypothyroidism Patients

Compared with SLE-S-hypothyroidism group, the average age of SLE-C-hypothyroidism group was older (42.21 ± 14.25 y vs. 36.67 ± 12.94 y, *P*=0.031), and SLE-C-hypothyroidism group had higher level of ESR (55.02 ± 33.10 mm/h vs. 33.21 ± 31.48 mm/h, *P*=0.001), creatinine (85.88 ± 86.00 mmol/L vs. 59.41 ± 22.21 mmol/L, *P*=0.014), 24h urine protein (2.24 ± 3.19 mg/d vs. 0.84 ± 1.44 mg/d, *P*=0.002) and lower level of C3 (0.63 ± 0.31 g/l vs. 0.78 ± 0.37 g/l, *P*=0.024), and albumin (29.52 ± 8.24 g/l vs. 36.05 ± 7.05 g/l, *P*<0.001). The prevalence of thyroid autoantibody (52.60% vs. 31.50%, *P*=0.026) and cardiac insufficiency (14.10% vs. 1.90%, *P*=0.027) were higher in SLE-C-Hypothyroidism group than in SLE-S-Hypothyroidism group (**Table 3**).

**Table 3:** Comparison of serological and clinical profiles in SLE with C-hypothyroidism and SLE with S-hypothyroidism.

	SLE-C-Hypothyroidism	SLE-S-Hypothyroidism	*P*
**Epidemiological data**			
N	78	54	
Age (y) M ± SD	42.21 ± 14.25	36.67 ± 12.94	**0.031**
Female n/N (%)	73/78 (93.60)	51/58 (94.4)	>0.999
**Serological profile**			
ESR (mm/h) M ± SD	55.02 ± 33.10	33.21 ± 31.48	**0.001**
C3 (g/l) M ± SD	0.63 ± 0.31	0.78 ± 0.37	**0.024**
Low C4 n/N (%)	35/64 (54.70)	20/45 (44.40)	0.391
ANA n/N (%)	56/56 (100.00)	37/38 (97.40)	0.404
Anti-dsDNA n/N (%)	25/56 (44.60)	19/40 (47.50)	0.945
Anti-SSA n/N (%)	23/50 (46.00)	23/39 (59.00)	0.317
Anti-SSB n/N (%)	5/50 (10.00)	2/39 (5.10)	0.461
Anti-SM n/N (%)	15/49 (30.60)	8/34 (23.50)	0.646
Thyroid autoantibody n/N (%)	41/78(52.60)	17/54 (31.50)	**0.026**
Creatinine (mmol/L) M ± SD	85.88 ± 86.00	59.41 ± 22.21	**0.014**
24 h urine protein (mg/d) M ± SD	2.24 ± 3.19	0.84 ± 1.44	**0.002**
Albumin (g/l) M ± SD	29.52 ± 8.24	36.05 ± 7.05	**<0.001**
Triglycerides (mmol/l) M ± SD	2.82 ± 1.53	2.07 ± 1.37	0.055
HDL (mmol/l) M ± SD	1.13 ± 0.47	1.04 ± 0.38	0.422
Diastolic pressure (mmHg) M ± SD	81.82 ± 14.91	81.56 ± 12.29	0.918
**Organic damage**			
Treatment **^a^** n/N (%)	30/78 (38.50)	24/54 (44.40)	0.612
Liver abnormality n/N (%)	19/78 (25.40)	14/54 (25.90)	>0.999
Serositis n/N (%)	15/78 (19.20)	8/54 (14.80)	0.671
LN n/N (%)	36/78 (46.20)	16/54 (29.60)	0.084
Hemolytic anemia n/N (%)	3/78 (3.80)	2/54 (3.70)	>0.999
Cardiac insufficiency n/N (%)	11/78 (14.10)	1/54 (1.90)	**0.027**
ILD n/N (%)	7/78 (9.00)	5/54 (9.30)	>0.999
PH n/N (%)	3/78 (3.80)	4/54 (7.40)	0.443
NPSLE n/N (%)	8/78 (5.30)	1/54 (1.90)	0.081

Bold values are statistical differences p < 0.05.

### Comparison Study of Laboratory and Clinical Variables in SLE-AH Patients and SLE-NAH Patients

Compared with SLE-NAH group, SLE-AH group had a lower creatinine level (58.15 ± 26.29 mmol/L vs. 87.58 ± 85.86 mmol/L, *P*=0.008) and a higher albumin level (34.25 ± 7.24 g/l vs. 30.61 ± 8.90 g/l, *P*=0.015). The treatment of glucocorticoids/immunosuppressants (0% vs. 73.00%, *P*<0.001) and the prevalence of LN (25.90% vs. 50.00%, *P*=0.008) was lower in SLE-AH group than in SLE-NAH group (**Table 4**).

**Table 4:** Comparison of serological and clinical profiles in SLE-AH and SLE-NAH groups.

	SLE-AH	SLE-NAH	*P*
**Epidemiological data**			
N	58	74	
Age (y) M ± SD	39.53 ± 14.22	40.26 ± 14.90	0.778
Female n/N (%)	57/58 (98.30)	67/74 (90.50)	0.078
**Serological profile**			
ESR (mm/h) M ± SD	52.29 ± 38.19	41.66 ± 30.17	0.112
C3 (g/l) M ± SD	0.73 ± 0.34	0.66 ± 0.35	0.263
Low C4 n/N (%)	22/48 (45.80)	33/61 (54.10)	0.507
ANA n/N (%)	38/38 (100.00)	55/56 (98.20)	>0.999
Anti-dsDNA n/N (%)	13/26 (33.30)	31/57 (54.40)	0.068
Anti-SSA n/N (%)	16/23 (41.00)	30/50 (60.00)	0.118
Anti-SSB n/N (%)	3/39 (7.70)	4/50 (8.00)	>0.999
Anti-SM n/N (%)	10/35 (28.60)	13/48 (27.10)	>0.999
Creatinine (mmol/L) M ± SD	58.15 ± 26.29	87.58 ± 85.86	**0.008**
24 h urine protein (mg/d) M ± SD	1.41 ± 2.39	1.87 ± 2.93	0.389
Albumin (g/l) M ± SD	34.25 ± 7.24	30.61 ± 8.90	**0.015**
**Organic damage**			
Treatment n/N (%)	0/58 (0)	54/74 (73.00)	**<0.001**
Liver abnormality n/N (%)	14/58 (24.10)	19/74 (25.70)	>0.999
Serositis n/N (%)	13/58 (22.40)	10/74 (13.50)	0.268
LN n/N (%)	15/58 (25.90)	37/74 (50.00)	**0.008**
Hemolytic anemia n/N (%)	4/58 (6.90)	1/74 (1.40)	0.168
Cardiac insufficiency n/N (%)	4/58 (6.90)	8/74 (10.80)	0.637
ILD n/N (%)	4/58 (6.90)	8/74 (10.80)	0.637
PH n/N (%)	3/58 (5.20)	6/74 (5.40)	>0.999
NPSLE n/N (%)	3/58 (5.20)	6/74 (8.10)	0.731

Bold values are statistical differences p < 0.05.

### Univariate and Multivariate Logistic Regression Analyses

On univariate analysis, differences in the treatment of glucocorticoids/immunosuppressants (COR, 0.280; 95%CL, 0.176-0.445; *P*<0.001), liver abnormality (COR, 2.536; 95%CL, 1.411-4.560; *P*=0.002), serositis (COR, 2.247; 95% CL, 1.149-4.392; *P*=0.008), LN (COR, 3.135; 95% CL, 1.886-5.212; *P*<0.001), and cardiac insufficiency (COR, 9.800; 95% CL, 2.156-44.544; *P*<0.001) were observed between SLE-hypothyroidism and SLE-euthyroidism groups (**Table 5**).

**Table 5:** Univariate and multivariate analyses of risk factors for hypothyroidism in SLE.

	COR (95CI%)	*P*	AOR (95%CI)	*P*
Treatment	0.280 (0.176-0.445)	**<0.001**	0.226 (0.135-0.377)	**<0.001**
Liver abnormality	2.536 (1.411–4.560)	**0.002**	2.410 (1.250–4.646)	**0.009**
Serositis	2.247 (1.149–4.392)	**0.018**		
LN	3.135 (1.886–5.212)	**<0.001**	3.591 (2.024–6.372)	**<0.001**
Cardiac insufficiency	9.800 (2.156–44.544)	**0.003**	8.410 (1.752–40.374)	**0.008**

AOR, adjusted odds ratio; LN, lupus nephritis.

Bold values are statistical differences p < 0.05.

On multivariate analysis, the treatment of glucocorticoids/immunosuppressants (AOR, 0.226; 95% CL, 0.135-0.377; *P*<0.001), liver abnormality (AOR, 2.410; 95% CL, 1.250-4.646; *P*=0.009), LN (AOR, 3.591; 95% CL, 2.024-6.372; *P*<0.001), and cardiac insufficiency (AOR, 8.410; 95% CL, 1.752-40.374; *P*=0.008) were associated with hypothyroidism (**Table 5**).

## Discussion

### The Prevalence of Hypothyroidism in SLE

In this study, SLE patients had a significantly higher prevalence of hypothyroidism than control group (15.40 vs. 6.10%, *P*<0.001), which was consistent with previous studies (16, 17). SLE patients also had a higher prevalence of thyroid autoantibody than control group (22.8% vs. 16.9%, *P*<0.001), which might lie in poly-autoimmunity and pleiotropy of nonspecific disease genes between SLE and AIT (18). Previous study considered that the higher prevalence of hypothyroidism in SLE might have relationship with the higher prevalence of AIT in SLE. In Alessandro Antonelli’s research, SLE patients had higher prevalence of both C-hypothyroidism and S-hypothyroidism than control group (19). However, in this study, there was a statistically significant difference only in the prevalence of C-hypothyroidism between SLE and control groups. The study of Alessandro Antonelli did not exclude patients with overlap syndrome, thyroid surgery, and thyroid-related medications during the population selection phase. This might lead to a higher prevalence of S-hypothyroidism in SLE patients. So, the prevalence of hypothyroidism in our control group was also lower than that in previous Chinese epidemiological study (6.10% vs. 13.95%).

The previous study revealed that the prevalence of hypothyroidism increased with aging and was more frequent in females, which was verified by the results of the control group in this study (**Figures 2A**, **3A**). We found that the prevalence of hypothyroidism raised with the increase of age. It might result from the reduction of thyroid activity due to decreased TSH concentrations and damnification of peripheral 5’-deiodinase (20). However, some demographic characteristics of patients with hypothyroidism complicated with SLE have changed. In this study, the prevalence of hypothyroidism was the highest in SLE patients aged 16-26 years old and gradually decreased with the increase of age. The early onset of SLE (20-40 years) might contribute to this phenomenon. SLE leads to the release of some cytokines and chemokines, and then thyroid is damaged after they reach thyroid tissue by circulation. The mortality rate in SLE patients is still three times higher than that in general population (6). With the increase of age, SLE patients have more complications, which led to a higher mortality rate and reduced prevalence of hypothyroidism in older SLE patients.

### The Relationship Among SLE, AIT, and Hypothyroidism

The increased prevalence of AIT in SLE is considered due to common genetic background and disordered immune environment in SLE. Previous studies suggested that SLE patients were prone to hypothyroidism due to the increased prevalence of AIT, which was inconsistent with our results. When SLE patients and the general population were combined with positive thyroid autoantibodies, there were slightly difference between the two groups (**Figure 4A**), which mainly came from TgAb+, rather than TPOAb+ (**Figure 4B**). When SLE patients and the general population were combined with negative thyroid autoantibodies, the prevalence of hypothyroidism between the two groups was significantly different. TPOAb can participate in the process of complement fixation and activation and inhibit the activity of thyroid peroxidase by binding to its active site and play an important role in the immune response of AIT (21). TgAb is an important marker of thyroid damage, which often occurs in circulation after thyroid fine needle puncture, thyroidectomy and subacute thyroiditis (22). In AIT patients, TPOAb and TgAb are both marker antibodies, but the sensitivity of TPOAb is much higher than TgAb. In this study, there was no significant difference in the prevalence of hypothyroidism between SLE group and control group when TPOAb was positive (TgAb negative). However, there was a significant difference in the prevalence of hypothyroidism between the two groups when TgAb was positive (TPOAb negative) (**Figure 4B**). This might indicate that the higher prevalence of hypothyroidism in SLE group was not related to AIT. When thyroid autoantibodies were negative, the prevalence of hypothyroidism in SLE group was significantly higher than that in control group. Excessive or low iodine intake is an important cause of non-autoimmune hypothyroidism. SLE patients and healthy people included in this study lived in Shandong Province. Study showed that iodine nutritional status in this region was at an appropriate level (23), which meant that the higher prevalence of hypothyroidism in SLE patients with TgAb-TPOAb- was not related to iodine intake. Whether thyroid autoantibodies were positive or negative, the elevated prevalence of hypothyroidism in SLE patients could not be completely explained by AIT and iodine intake. Therefore, we speculated that the pathogenesis of hypothyroidism in SLE patients might be different from hypothyroidism in the general population.

### Glucocorticoids/Immunosuppressants and Hypothyroidism

Glucocorticoids and immunosuppressants, the most useful drug for SLE, have anti-inflammatory and immunosuppressive effects. Those effects of glucocorticoids act in two distinct ways. One is the genomic way that includes two different processes, namely transactivation and transrepression (24), ultimately lowering the expression of proinflammatory cytokines such as TNF-α, IL-8, IL-6 or increasing the expression of anti-inflammatory proteins such as IL-10. Glucocorticoids can also act by non-genomic mechanisms, which produces a rapid and potent anti-inflammatory action (25). In this study, patients who had a history of using glucocorticoids/immunosuppressants were all TgAb-TPOAb-. Interestingly, we found that the prevalence of hypothyroidism was negatively related to the treatment of glucocorticoids. Glucocorticoids/immunosuppressants could inhibit lymphocyte activity and reduce antibody production, which might lead to the decrease of prevalence of hypothyroidism in SLE. On the other hand, the treatment of glucocorticoid/immunosuppressant might be related to patient compliance. Patients with better compliance in this study might have higher rate of glucocorticoid/immunosuppressant treatment and better disease control. SLE patients with hypothyroidism had severer systemic organic damage, higher disease activity (higher ESR, lower C3 and C4), and lower treatment rate of glucocorticoids/immunosuppressants (**Table 2**). These results revealed that SLE patients with hypothyroidism were in a severer state. Whether poor control of SLE is an important cause of hypothyroidism still needs to be proved by further prospective clinical cohort studies.

### LN and Hypothyroidism

In this study, we observed there were more LN patients in the hypothyroidism group. This suggested that LN could be related to hypothyroidism. In multiple logistic regression models, LN was retained as an independent risk factor for hypothyroidism. Kidney disease can result in hypothyroidism, both clinical and subclinical (26, 27). Thyroid hormone urinary loss, malnutrition, and iodine depletion may be the causes of hypothyroidism (28). For the SLE patients in this study, 24h urine protein was significantly higher in the SLE-hypothyroidism group. Besides, severer hypoalbuminemia was observed in those with hypothyroidism. These all indicated hypothyroidism might be related to thyroid hormone urinary loss in LN. Both anti-dsDNA antibodies and complement levels were considered as the signs of activities for patients with LN. In the present study, more patients with lower levels of C3 and C4 were observed in the hypothyroidism group, which indicated that hypothyroidism was not only associated with thyroid urinary loss but also proliferative nephritis.

### Liver Abnormality and Hypothyroidism

The gastrointestinal system is another susceptible system in SLE, in which liver abnormality is one of the most common manifestations. It was reported that 3–8% of SLE patients had slightly elevated liver enzymes (29). A retrospective study also revealed that 47 SLE patients (9.3%) had a liver abnormality, and active SLE was associated with a higher prevalence of liver abnormality than inactive SLE (11.8% vs. 3.2%, *p*<0.05) (30). As the center of human metabolism, liver plays an important role in thyroid hormone activation, inactivation, transport, and metabolism. As the target organ of thyroid hormones, hepatic cells can express thyroid hormone receptors. Meanwhile, thyroid hormones can affect lipid deposition in the liver by regulating oxidative stress, lipid metabolism, thyroid hormone receptor expression, mitochondrial function, etc (31). Hypothyroidism can lead to reduced decomposition of low-density lipoprotein and triglycerides, slow oxidation of cholesterol, and excessive lipid deposition in the liver (32). The serum thyroid hormone level can also reflect hepatic cells damage and hepatic lobular fibrosis of patients with liver diseases to some extent (33). In this study, SLE-hypothyroidism patients had a higher prevalence of liver abnormality than SLE-euthyroidism patients. SLE patients with simultaneous drug-induced liver injury/viral hepatitis/autoimmune hepatitis/cirrhosis were excluded in the data processing stage. The SLE-hypothyroidism group had a higher level of triglyceride than the SLE-euthyroidism group (2.55 ± 1.51 mmol/l vs. 1.64 ± 0.84 mmol/l, *P*<0.001)) and a lower level of high-density lipoproteins (1.10 ± 0.43 mmol/l vs. 1.34 ± 0.48 mmol/l, *P*=0.004)). We speculated that hypothyroidism might increase the prevalence of liver abnormality by affecting liver lipid metabolism in SLE patients.

### Cardiac Insufficiency and Hypothyroidism

Coronary atherosclerotic heart disease (CHD) is the most common SLE-related cardiovascular disease (CVD), which can cause cardiac insufficiency. A recent prospective study reported that during the five-year follow-up period, SLE patients (32%) had a higher rate of carotid atherosclerosis compared with general population (4%). Thyroid hormones also have extensive effects on the cardiovascular system. They act in three ways: 1) regulating the expression of target genes by binding to nuclear receptors of myocardial cells; 2) regulating ion channels that act on myocardial cell membrane through extranuclear nongenome; 3) influencing cardiovascular hemodynamics, cardiac filling and contractile capacity through thyroid hormones. Studies showed that diastolic blood pressure alone increased in SLE patients with hypothyroidism (34), but another study found that systolic and diastolic blood pressure increased simultaneously (35). The increased blood pressure in these hypothyroid patients might result from the increase of systemic vascular resistance, endothelial dysfunction, and low renin level. Hypothyroidism could also increase the risk of CVD by affecting lipid metabolism. A prospective cohort study showed that the risk of morbidity and mortality of CVD in SLE patients with subclinical hypothyroidism increased significantly. In this study, the prevalence of cardiac insufficiency in the SLE- hypothyroidism group was higher than that in the SLE-euthyroidism group. In multivariate logistic regression, cardiac insufficiency was an independent risk factor for hypothyroidism in SLE patients. Compared with SLE-euthyroidism patients, patients with SLE-hypothyroidism had higher diastolic blood pressure (81.71 ± 13.86 mmHg vs. 78.30 ± 12.62 mmHg, *P*=0.026), higher level of triglyceride (2.55 ± 1.51 mmol/l vs. 1.64 ± 0.84 mmol/l, *P*<0.001). According to the above results, we speculated that hypothyroidism might impair cardiac function by affecting diastolic blood pressure and lipid metabolism in SLE patients.

### The Interaction Between SLE and Hypothyroidism

**Figure 5:**
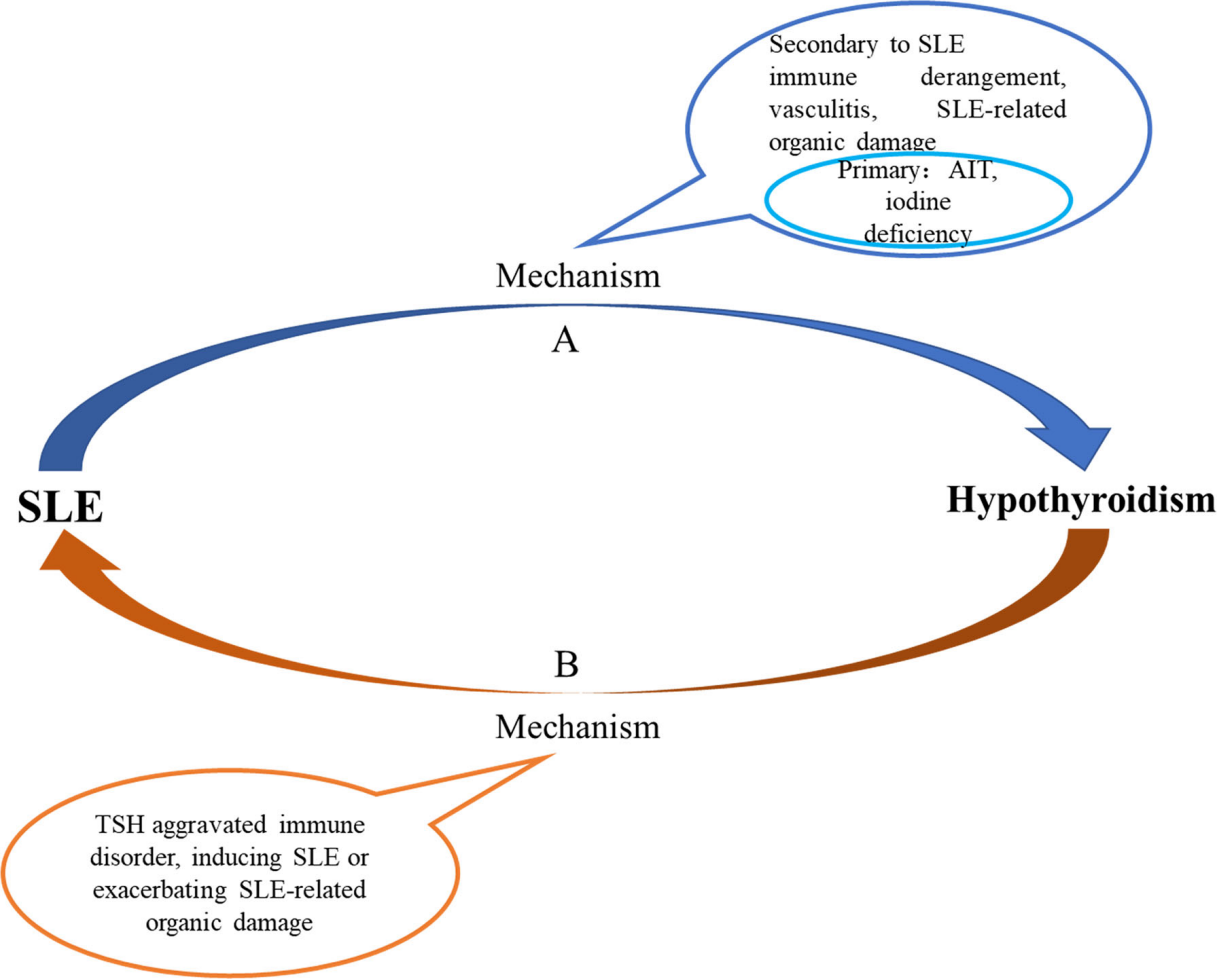
Potential mechanism of interaction between SLE and hypothyroidism.

With the increase of age, the prevalence of hypothyroidism in SLE gradually decreased; the prevalence of hypothyroidism in SLE patients with TgAb+TPOAb- or TgAb-TPOAb- was higher than that in the controls with TgAb+TPOAb- or TgAb-TPOAb-; compared with SLE-S-hypothyroidism patients, SLE-C-hypothyroidism patients were more severe (higher ESR and lower C3); lupus kidney, liver abnormality, and cardiac insufficiency were independent risk factors of hypothyroidism. All those characteristics of hypothyroidism in SLE were different from those in the general population, which indicated that the cause of hypothyroidism in SLE might be distinct from that in the general population. Previous studies found that thyroid hormone autoantibodies (THAb) were the first thyroid autoantibody in early thyroid injury, and SS or rheumatoid arthritis patients with THAb would develop to hypothyroidism within 10 years, suggesting that patients with rheumatic diseases had been accompanied by thyroid damage before hypothyroidism (36). Based on the above evidence, we hypothesized that the cause of hypothyroidism in SLE might include two types (**Figure 5A**). The first one, like the general population, was hypothyroidism due to AIT or iodine deficiency. The second might be a special hypothyroidism secondary to SLE. SLE disease itself and SLE-related organic damage could cause thyroid structural damage. When SLE occurred, the systemic immune response was activated. After reaching the thyroid *via* blood circulation, cytokines and chemokines released by immune cells led to inflammatory cell infiltration and thyroid injury, which further aggravated the immune response, and ultimately caused hypothyroidism. Immune complex deposited in the small and medium vascular walls led to inflammation, necrosis, and thrombosis in the vascular wall, and caused ischemia and hypoxia of the blood supply tissue, finally resulting in necrosis and dysfunction. This process was called vasculitis, which was the main pathological change of SLE. Thyroid, as a blood-rich endocrine gland, might also have hypothyroidism due to vasculitis. What’s more, when SLE patients had related organic damage (such as LN and liver abnormality), the binding of thyroid hormones and related carrier proteins and the excretion of thyroid hormones would be affected, which might also lead to hypothyroidism. We speculated that SLE could lead to hypothyroidism and inversely, hypothyroidism might also have an impact on the occurrence and development of SLE. Studies revealed that TSH has pro-inflammatory effect (37). The results of this study also showed that compared with SLE-euthyroidism patients, SLE- hypothyroidism patients had higher disease activity (higher ESR, lower C3 and C4) and severer organic damage, indicating that hypothyroidism might aggravate the immune disorder of SLE. However, more rigorous prospective cohort studies and pathological evidence were needed to prove our conjecture.

There were several strengths in this study. This is the first research focused on the relationship between hypothyroidism and organic damage in SLE. Second of all, comparatively speaking, the sample size of this study was large (856 SLE patients), which can obviously reduce the selection bias. What’s more, we selected 856 age- and sex-matched controls and acquired the history of the use of glucocorticoids and immunosuppressants within three months before hospitalization, increasing reliability of this research. However, this study also had several limitations. Firstly, this cohort study was a single-center study, and a multi-center study would provide more convincing data. Second, this study was a retrospective study with some important clinical data missed, such as the SLE disease activity score and cumulative dose of glucocorticoids/immunosuppressants. Finally, this study was a cross-sectional study and was unable to determine the causal relationship between organic damage and hypothyroidism.

## Conclusion

The prevalence of hypothyroidism in SLE patients was higher than that in controls and decreased with age. The results suggested that young SLE patients combined with LN, liver abnormality and cardiac insufficiency were at higher risk of hypothyroidism. According to the results of this study, we speculated that SLE might have impact on thyroid, and SLE might be one of the causes of hypothyroidism.

## Data Availability Statement

The raw data supporting the conclusions of this article will be made available by the authors, without undue reservation.

## Ethics Statement

The studies involving human participants were reviewed and approved by the Biomedical Research Ethics Committee of Shandong Provincial Hospital. (NO. SWYX2020-187). Written informed consent for participation was not required for this study in accordance with the national legislation and the institutional requirements.

## Author Contributions

All authors contributed to the study’s conception and design. Data collection and analysis were performed by JN, YW, LG, HL, LZ. The first draft of the manuscript was written by JN, JL, and HZ. All authors commented on previous versions of the manuscript. All authors read and approved the final manuscript.

## Funding

This work was supported by the Natural Science Foundation of China (81670721).

## Conflict of Interest

The authors declare that the research was conducted in the absence of any commercial or financial relationships that could be construed as a potential conflict of interest.

## Publisher’s Note

All claims expressed in this article are solely those of the authors and do not necessarily represent those of their affiliated organizations, or those of the publisher, the editors and the reviewers. Any product that may be evaluated in this article, or claim that may be made by its manufacturer, is not guaranteed or endorsed by the publisher.
